# Non-native populations of an invasive tree outperform their native conspecifics

**DOI:** 10.1093/aobpla/plw071

**Published:** 2016-10-13

**Authors:** Heidi Hirsch, Isabell Hensen, Karsten Wesche, Daniel Renison, Catherina Wypior, Matthias Hartmann, Henrik von Wehrden

**Affiliations:** 1 Department of Botany and Zoology, Centre for Invasion Biology, Stellenbosch University, Matieland, South Africa; 2 Institute of Biology/Geobotany and Botanical Garden, Martin Luther University Halle-Wittenberg, Halle, Germany; 3 German Centre for Integrative Biodiversity Research (iDiv) Halle-Jena-Leipzig, Leipzig, Germany; 4 Senckenberg Museum of Natural History Goerlitz, Am Museum 1, 02826 Goerlitz, Germany; 5 Centro de Ecología y Recursos Naturales Renovables – Dr. Ricardo Luti (CERNAR – FCEFyN – UNC) and Instituto de Investigaciones Biológicas y Tecnológicas (IIByT – CONICET – UNC), Av. Vélez Sarsfield 1611, Córdoba X5016GCA, Argentina; 6 Herbarium PRC & Department of Botany, Charles University in Prague, Benátská 2, 12801 Praha, Czech Republic; 7 Institute of Ecology/Faculty of Sustainability, Centre of Methods, Leuphana University, Scharnhorststraße 1, 21335 Lueneburg, Germany; 8 Research Institute of Wildlife Ecology, Savoyen Strasse 1, 1160 Vienna, Austria

**Keywords:** Biomass, genetic shift, greenhouse, post-germination traits, shoot-root ratio, *Ulmus pumila*

## Abstract

Introduced plants often face new environmental conditions in their non-native ranges. To become invasive, they need to overcome several biotic and abiotic filters that may trigger adaptive changes in life-history traits, like post-germination processes. Such early life cycle traits may play a crucial role in the colonization and establishment success of invasive plants. As a previous study revealed that seeds of non-native populations of the woody Siberian elm, *Ulmus pumila*, germinated faster than those of native populations, we expected growth performance of seedlings to mirror this finding. Here, we conducted a common garden greenhouse experiment using different temperature and watering treatments to compare the biomass production of *U. pumila* seedlings derived from 7 native and 13 populations from two non-native ranges. Our results showed that under all treatments, non-native populations were characterized by higher biomass production and enhanced resource allocation to aboveground biomass compared to the native populations. The observed enhanced growth performance of non-native populations might be one of the contributing factors for the invasion success of *U. pumila* due to competitive advantages during the colonization of new sites.

## Abstract (precis)

We compared the seedling growth performance of native and non-native Siberian elm populations in a common greenhouse experiment to gain knowledge about possible changes in life history traits in populations of non-native woody species. Our results showed that non-native Siberian elm populations are characterized by an enhanced early life cycle performance which likely contributes to its invasion success. This study contributes to a better understanding of the still little-known invasion processes of woody non-native species.

## Introduction

Organisms that become successful invaders after being introduced into non-native ranges must pass several selective biotic or abiotic filters that may trigger rapid evolutionary change (Novak 2007; Prentis *et al.* 2008). Such change can contribute to their invasion success, for example, by altering phenotypic traits (Bossdorf *et al.* 2005) that enhances tolerance to biotic or abiotic conditions (Maron *et al.* 2004; Abhilasha and Joshi 2009; Leiblein-Wild *et al.* 2014), or increasing early life cycle traits like germination or seedling growth (Blair and Wolfe 2004). Early life cycle trait performances are of great importance for the spread and establishment of introduced populations in a new range. For example, fast germination and growth of non-native plants can provide competitive advantages over resident species due to an earlier use of limited resources (Milbau *et al.* 2003; Barney *et al.* 2009). A shift in growth strategies in non-native populations often indicates enhanced efficiency of resource allocation (Zou *et al.* 2007).

To determine if phenotypic changes in non-native populations are caused by genetic processes or by phenotypic plasticity, it is important to compare the performance of native source populations as well as non-native populations in a common environment (Kawecki and Ebert 2004; Erfmeier and Bruelheide 2005; van Kleunen *et al.* 2010). In this regard, genetic diversity is of special interest due to its role for facilitating evolutionary responses of populations confronted with environmental changes (Reed and Frankham 2003). It has been found that non-native populations are often characterized by reduced genetic diversity compared with native populations due to bottlenecks during introduction (Sakai *et al.* 2001, Hirsch *et al.* 2011). Despite this, rapid evolution of non-native species may occur even when experiencing bottleneck events (Dlugosch and Parker 2008; Schrieber and Lachmuth 2016; Zenni *et al.* 2016a, b, this issue). Some studies, however, provide evidence that reduced genetic diversity is not always the rule for non-native populations, and that genetic diversity can be maintained by different mechanisms dependent on introduction history (multiple vs. single introductions), native range genetic structure and propagule pressure (Petit *et al.* 2004; Prentis *et al.* 2008; Le Roux *et al.* 2011; Mandák *et al.* 2013). For example, inter- or intraspecific hybridization can lead to similar or increased diversity levels compared with source populations and can generate gene combinations which might be better adapted or more tolerant to novel environmental conditions (Ellstrand and Schierenbeck 2000; Dormontt *et al.* 2011).

Although only a small proportion of the world's woody plant species is currently considered invasive, the impacts of these invasions are increasing worldwide (Rejmánek and Richardson 2013). To date, only few consistent traits explaining invasion success of woody plants have been found (Moles *et al.* 2012, Richardson *et al.* 2014) which highlights the need for more detailed knowledge about factors contributing to the invasion success of trees or shrubs to predict and prevent further invasion processes. Trait dynamics of trees are particularly poorly understood in such species due to their long lifespans and generation times (Zenni *et al.* 2016a,b, this issue). Here we focus on differences in post-germination traits between native and invasive populations of the Siberian Elm, *Ulmus pumila* (Ulmaceae). This species was introduced in several regions outside its native range and is today regarded as naturalized or invasive in most regions where it has been introduced. Its invasion success can partly be explained by inter- and intraspecific hybridization, leading to high genetic diversity in the corresponding non-native populations (Cogolludo-Agustín *et al.* 2000; Zalapa *et al.* 2010; Hirsch *et al.* unpubl. data). In a previous study, we showed that non-native *U. pumila* populations from the Southwestern U.S. are characterized by increased germination rates compared to native populations from China (Hirsch *et al.* 2012). Based on genetic data, it is highly probable that admixture supported this observed shift in the germination traits of non-native Siberian elm populations from the Southwestern U.S. (Hirsch *et al.* unpubl. data). Several studies have shown that differences in germination characteristics can also be mirrored in post-germination traits (Donohue *et al.* 2010). We thus performed a common greenhouse experiment with non-native and native *U. pumila* populations to test if post-germination traits show differences between native and invasive populations resulting in an enhanced growth performance of non-native individuals from two invasion regions. We included different temperature and watering treatments to simulate a wide range of environmental conditions. In particular, we tested two hypotheses: (1) non-native populations of *U. pumila* will have increased above- and belowground biomass production compared to native populations; and (2) that this enhanced growth performance in non-native populations is achieved through changes in above- and belowground biomass allocation, and thus more efficient resource usage.

## Methods

### Study species

The Siberian elm, *U. pumila*, is a diploid tree native to northern and eastern China, central Mongolia, as well as southern Russia where it can grow in various topographies like slopes, valleys, and plains (Wu *et al.* 2003; von Wehrden *et al.* 2009; Wesche *et al.* 2011). *Ulmus pumila* is characterized by fast growth, and it can persist under harsh climatic conditions such as long summer droughts and cold winters (Wu *et al.* 2003; USDA and NRCS 2011). Its growth performance and a high tolerance to the Dutch Elm Disease led to its wide distribution and its use in breeding programs outside the native range (Leopold 1980; Mittempergher and Santini 2004). Today, the Siberian elm is considered as naturalized or invasive in the U.S., Canada (Kartesz 2011; USDA and NRCS 2011), Mexico (Todzia and Panero 1998), Argentina (Mazia *et al.* 2001; Zalba and Villamil 2002), Spain (Cogolludo-Agustín *et al.* 2000), Estonia, Australia and the European part of Russia (NOBANIS 2012).

### Sampling scheme

**Figure 1:**
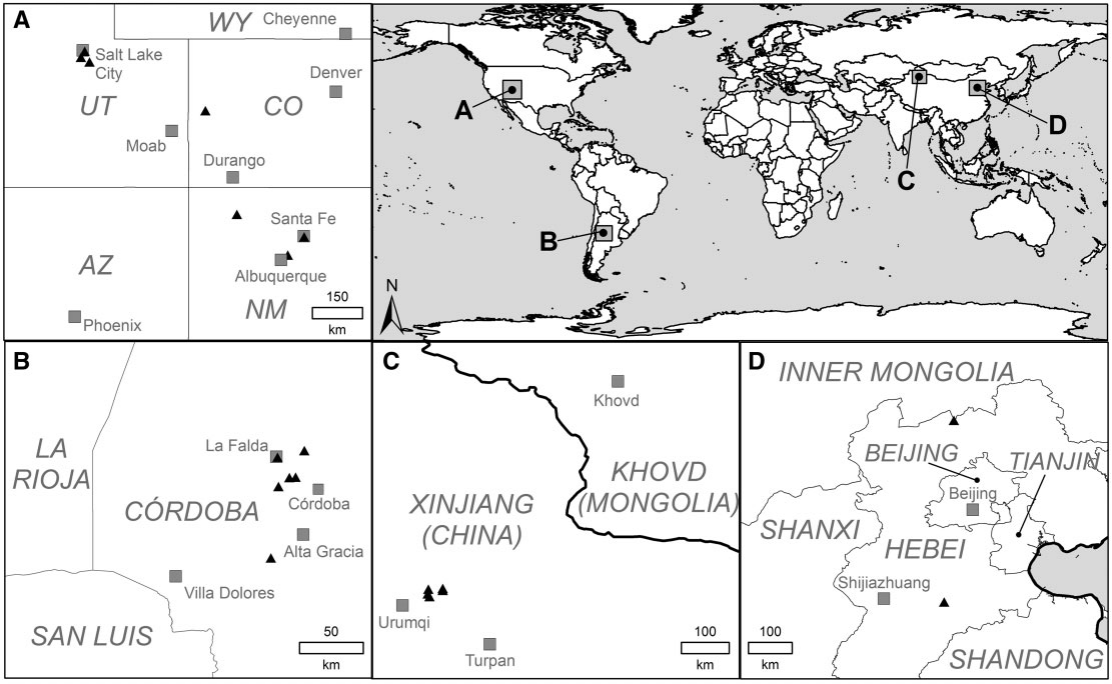
Sampled localities of *Ulmus pumila* populations in the non-native ranges (A: U.S. and B: Argentina) and in the native range (C and D: China). Population locations are indicated by gray triangles. AZ , Arizona; CO, Colorado; NM, New Mexico; UT, Utah; WY, Wyoming.

We included seven populations from the Chinese native range, seven populations from the North American and six populations from the Argentinean non-native ranges (Fig. 1) **[see Supporting Information–Table S1]**. To minimize the chances of sampling inter-specific hybrid individuals, we chose only non-native populations located in regions where no other elm species capable of hybridizing with *U. pumila* occurred. A genetic study using microsatellite markers showed that the non-native populations in these regions are rather characterized by intra-specific hybridization and standing genetic diversity comparable to native populations of *U. pumila* (Hirsch *et al.* unpubl. data). In the native range, we focussed on populations from northern parts of China because these regions seem to be the most probable source regions of at least the North American non-native populations (Webb 1948; Leopold 1980). Mature seeds from China were collected in 2009 and from Argentina and the U.S. in 2010. At least, 15 trees per population were sampled and seeds were pooled within populations. Where seeds had already been shed, they were collected from the ground at different locations across the population to obtain a representative mixed sample of the corresponding population. To maintain seed viability, seed material was stored in sealed plastic bags at 4 °C following Grover *et al.* (1963).

### Growth experiment

Common garden experiments were initiated in January 2011 and were performed in a completely randomized design. For germination, we used eight replicates per population and a temperature treatment. Each replicate contained 20 seeds placed on filter paper in standard Petri dishes. The dishes were filled with de-ionized water to keep the seeds permanently moist. The germination was performed in RUMED Light Thermostats germination chambers (Type 1301; Rubarth Apparate GmbH, Laatzen, Germany) under two temperature treatments (20 °C/10 °C and 32 °C/20 °C) with a photoperiod of 12 h cold white light (1200 Lux) and 12 h darkness. Eight randomly chosen seedlings per population were planted into 1.5 L pots filled with a standardized amount of soil substrate (substrate “TS 3” with recipe number 404 supplied by Klasmann-Deilmann, Geeste, Germany). Age of seedlings within and between the different treatments differed by less than 5 days (minimum age: 3 days) and all seedlings were planted at the same day to minimise the effect of germination date on the results of the experiment. Further, only seedlings of similar size where selected to minimize maternal effects. Individuals were assigned to controlled greenhouse cabinets with alternating temperatures of either 20 °C/10 °C or 30 °C/20 °C (day/night). In this context, seeds which germinated under the lower temperature treatment were assigned to the lower temperature conditions in the greenhouse and vice versa. Additional illumination of sufficient intensity to allow growth was used during the whole growth experiment to provide a day length of 12 h. After an establishment period of 24 days with regular watering, the treatment with water levels of 90, 70 and 50 % (gravimetric percentages of the soil water holding capacity), hereafter referred to as wet, medium and dry, respectively, was applied. Every second day, gravimetrically determined water loss was adjusted and pots were re-randomized every 2 weeks within each greenhouse cabinet. To avoid block effects between cabinets all non-temperature and non-watering related conditions were equivalent and constant (e.g. cabinets located at the same side of the greenhouse, homogeneous illumination and humidity).

Resulting from the design outlined above, the initial experimental conditions included 960 individuals (20 populations * 2 temperature treatments * 3 water treatments * 8 replicates; Table 1). After 10 weeks of water treatment, all individuals were harvested by carefully removing the plants from the pots. The roots where gently cleaned from all attached soil particles using a root washing table. Following drying at 80 °C for 48 h, above- and belowground biomass was weighed for each plant using a high precision balance (Sartorius AG, Goettingen, Germany; model La5200d; readability 0.001 g).

**Table 1.** Number of *Ulmus pumila* individuals (*N*_ind_) per range at the beginning and at the end of the greenhouse experiment. The starting number consists of the number of populations tested for the corresponding range (*N*_pop_) * 2 temperatures * 3 water treatment levels * 8 replicates. The difference between the starting number and the number of individuals at the end of the experiment characterizes the number of individuals which did not survive the experiment (*N*_ind_dead_).

Range	*N*_pop_	*N*_ind_ at the beginning of the experiment	*N*_ind_ at the end of the experiment	*N*_ind_dead_
China (native)	7	336	305	31
Argentina (non-native)	6	288	273	15
U.S. (non-native)	7	336	332	4
Sum	20	960	910	50

### Statistical analyses

All statistical analyses were performed in the R statistical environment (version 3.2.2; R Core Team 2015). Biomass parameters were square root transformed to better approximate normality. We applied generalized linear mixed models with Gaussian error distribution to test if biomass production differs between ranges as well as temperature and water treatments (package lme4 version 1.1-10; Bates *et al.* 2015). Populations nested within range were included as random effect and backward elimination of non-significant fixed effects (*P* > 0.05) was used as criterion for model selection. Significances were derived by applying Wald Chi-Square tests (package car version 2.0-25; Fox and Weisberg 2011). Post-hoc analysis of significant interactions was performed using the R package phia (version 0.2-0; De Rosario-Martínez 2015). The same procedures were chosen for log-transformed shoot-root ratios (dry aboveground biomass divided by dry belowground biomass) as response variable. We considered this approach because biomass allocation patterns can help to gain a more detailed knowledge about how plants react towards different environmental conditions (e.g. Wilson 1987; Padilla *et al.* 2013).

## Results

Fifty individuals did not survive the experiment, and of these, more than 60 % were from native range populations (Table 1). Consequently, biomass was harvested from a total of 910 individuals (Table 1).

**Figure 2:**
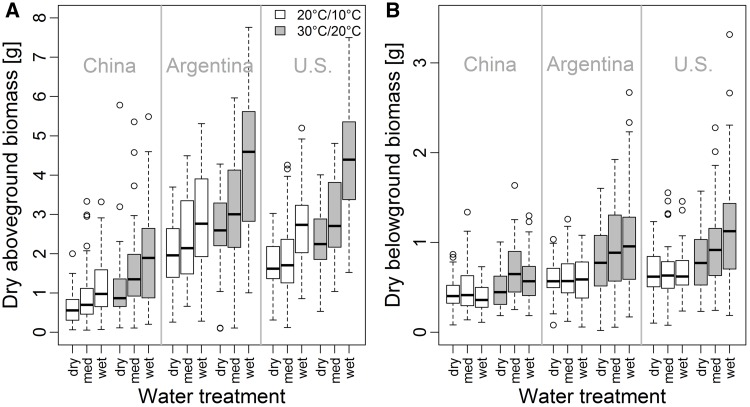
Dry aboveground (A) and dry belowground (B) biomass of native and non-native *Ulmus pumila* populations by temperature and water treatments (med = medium). For a statistical analysis of the data see Table 2.

**Figure 3:**
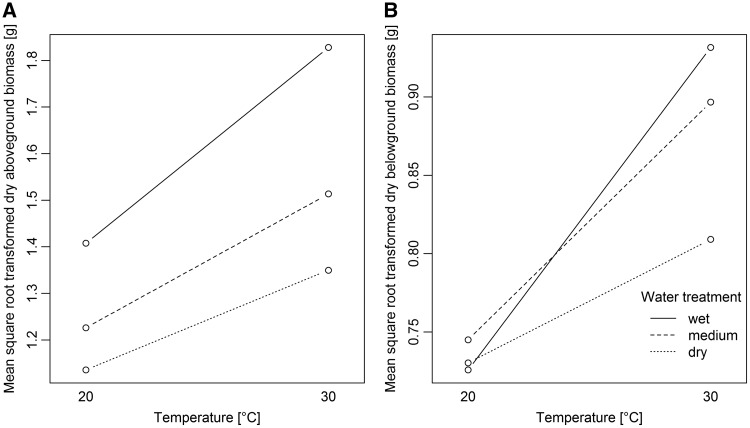
Interaction plots of mean dry aboveground biomass (A) and mean dry belowground biomass (B) of *Ulmus pumila* in response to the temperature and water treatments.

Aboveground biomass was significantly higher under the warmer temperature treatment, the medium as well as the wet water treatment, and in non-native populations (Table 2 and Fig. 2A). Further, we found a significant interaction between temperature and water treatment (Table 2). The post-hoc analysis of this interaction revealed that the warmer temperature treatment had a stronger positive effect for the aboveground biomass production under the wet water treatment than under the medium and dry water treatments (*χ*^2 ^ = ^ ^5.59, d*f*  =  1, adjusted *P*  =  0.036) and to the dry water treatment (*χ*^2 ^ = ^ ^15.28, d*f*  =  1, adjusted *P* < 0.001; Fig. 3A). The medium and the dry water treatments showed a similar response to the temperature treatments (*χ*^2 ^ = ^ ^2.46, d*f*  =  1, adjusted *P* > 0.05; Fig. 3A).

**Table 2.** Analysis of variance (type III) results comparing the minimal generalized linear mixed models. The results show the influence of the temperature and water treatment as well as the population origins (range) on the aboveground and belowground biomass production as well as on the biomass ratio (d*f *,  degrees of freedom; temp.,   temperature; treatm.,  treatment). Rows with no entries characterize interactions that were removed during the model reduction process.

Source of variance	Aboveground biomass				Belowground biomass				Biomass ratio		
	***χ***^2^ Wald statistic	d*f*	*P*		***χ***^2^ Wald statistic	d*f*	*P*		***χ***^2^ Wald statistic	d*f*	*P*
Intercept	103.17	1	<0.001		415.50	1	<0.001		7.00	1	0.008
Temp. treat.	30.56	1	<0.001		13.23	1	<0.001		58.90	1	<0.001
Water treat.	60.01	2	<0.001		1.03	2	0.596		144.61	2	<0.001
Range	41.93	2	<0.001		23.58	2	<0.001		29.88	2	<0.001
Temp. × water treat.	15.47	2	<0.001		18.17	2	<0.001				
Temp. treat. × range									36.61	2	<0.001
Water treat. × range									11.27	4	0.024
Temp. treat × water treat. × range											

Belowground biomass production was also significantly higher under warmer temperatures and for non-native populations (Table 2 and Fig. 2B). The water treatment showed a significant effect only in interaction with the temperature treatment (Table 2). This interaction reflects a similar reaction of belowground biomass production under medium and wet water treatment levels between the two temperature conditions (*χ*^2 ^ = ^ ^3.00, d*f*  =  1, adjusted *P* > 0.05; Fig. 3B). Belowground biomass production under dry conditions was less affected by the temperature treatment compared to the medium (*χ*^2 ^ = ^ ^6.42, d*f * =  1, adjusted *P*  =  0.023) and wet water treatment level (*χ*^2 ^ = ^ ^17.94, d*f*  =  1, adjusted *P* < 0.001; Fig. 3B).

**Figure 4:**
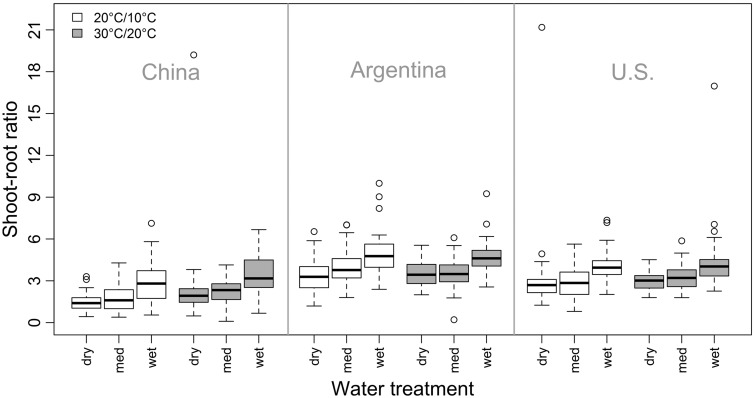
Shoot–root ratios of native and non-native *Ulmus pumila* populations in response to temperature and water treatments (med = medium). For a statistical analysis of the data see Table 2.

**Figure 5:**
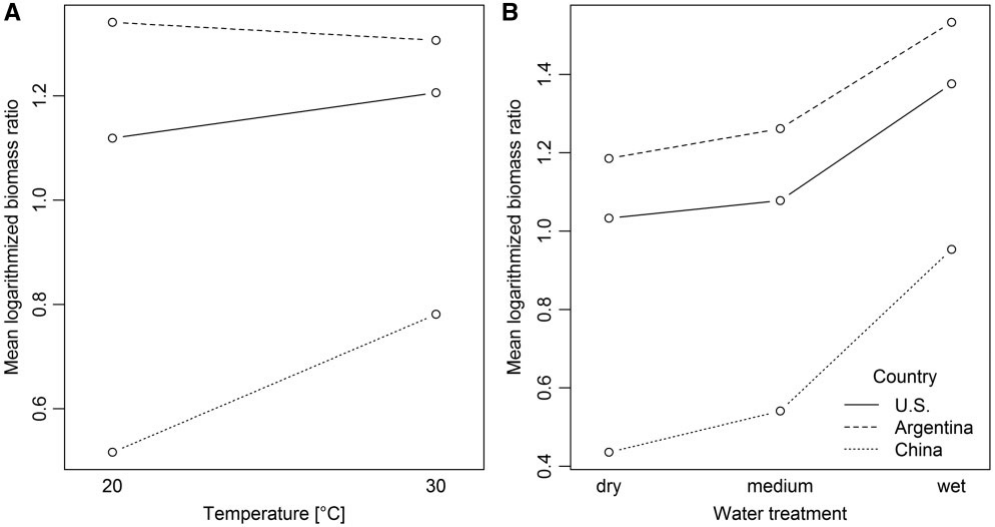
Interaction plots of mean shoot-root ratio in response to temperature (A) and water treatments (B).

Similar patterns were found for shoot-root ratio, which was significantly higher under warmer temperatures and for both non-native ranges (Table 2 and Fig. 4). However, a significant interaction between temperature treatment and range (Table 2) showed that the warmer temperature treatment had a stronger increasing effect on the shoot-root ratio of native populations compared to non-native populations from Argentina (*χ*^2 ^ = ^ ^35.51, d*f*  =  1, adjusted *P* < 0.001) and the U.S. (*χ*^2 ^ = ^ ^15.07, d*f*  =  1, adjusted *P* < 0.001; Fig. 5A). Both non-native ranges differed only slightly regarding their response to the water treatment (*χ*^2 ^ = ^ ^5.43, d*f*  =  1, adjusted *P* < 0.02; Fig. 5A). Shoot–root ratios were also significantly different between the water treatment levels, with lowest values under the dry water treatment and highest values under the wet water treatment (Table 2 and Fig. 4). Moreover, water treatment and range showed a significant interaction (Table 2). The shoot–root ratio of native populations was more positively affected by the water treatment than the shoot–root ratio of non-native Argentinean (*χ*^2 ^ = ^ ^8.17, d*f*  =  2, adjusted *P*  =  0.044) or non-native U.S. populations (*χ*^2 ^ = ^ ^8.46, d*f*  =  2, adjusted *P*  =  0.044; Fig. 5B). We found no differences between both non-native ranges within this interaction (*χ*^2 ^ = ^ ^0.26, d*f*  =  2, adjusted *P* > 0.05; Fig. 5B).

## Discussion

The greenhouse study supported our hypotheses that saplings of non-native populations of *Ulmus pumila* were characterized by increased above- and belowground biomass production combined with changes in above- and belowground biomass allocation across all water and temperature treatments compared to native range populations. Thus, enhanced seed germination rates of non-native Western U.S. populations compared with native populations (Hirsch *et al.* 2012) are reflected in an increased seedling performance of these populations. This result is in accordance with the assumption that a change of germination traits often involves a change of post-germination traits (Donohue *et al.* 2010). These characteristics in non-native populations might contribute to the invasion success of *U. pumila* because they promote advantages during the seedling establishment and colonization of new sites. For example, fast germination allows an earlier use of resources, while enhanced biomass production promotes competitiveness (Seiwa 2000; Weigelt *et al.* 2002). Blumenthal and Hufbauer (2007), by comparing native and non-native populations of 14 different invasive herbaceous plant species, showed that invasive species often evolve increased growth. However, they also found that this is only when introduced plants are not competing with natives. Thus, without experiments to compare the competitive ability of native and non-native *U. pumila* populations, we cannot draw conclusions on the full spectrum of environments where this shift in early life cycle traits might be beneficial. Nevertheless, our results show that the enhanced early life cycle performance may support rapid establishment and colonization of non-native Siberian elm populations, at least in non- or low-competitive environments (e.g. sites treated by mowing, burning or removal of trees and shrubs, natural sites where no or only few other trees or shrubs occur).

Data on shoot-root ratios showed that populations in both non-native ranges may have enhanced efficiency in resource allocation into aboveground biomass. A similar biomass allocation shift was found when comparing the growth performance of native and non-native *Phragmites australis* populations (Saltonstall and Stevenson 2007). These authors demonstrated that, when grown in high nutrient levels, non-native *P. australis* populations invest more in the shoot production than native populations and that this can explain its aggressive growth in the non-native range. Because we observed biomass allocation shifts in non-native populations across all treatments, we assume that this contributes towards an advantage in the establishment of new populations at different environmental conditions in the non-native ranges of *U. pumila*. The increased investment in aboveground biomass may indicate a competitive advantage over slower growing species since this allows a better use of resources (Gioria and Osborne 2014). Further, the higher allocation into belowground biomass of native Siberian Elm populations might indicate higher disturbance pressure by herbivory in this range where resource storage becomes an important strategy for survival (van der Maarel and Titlyanova 1989; Jia *et al.* 2010). In this context, it is often assumed that a shift in the biomass allocation and the overall growth performance of non-native populations is caused by a resource reallocation from defense mechanisms into vegetative growth due to a release of selection pressures (e.g. herbivores) as postulated by the EICA hypothesis (evolution of increased competitive ability; Blossey and Notzold 1995). However, more comparative experiments with respect to the response to herbivory and inter-specific competition are needed to test the predictions of the EICA hypothesis for the invasion of *U. pumila*. In contrast, it is also possible that the higher allocation into belowground biomass in native populations may indicate an adaptation to less favorable soil water availabilities in these localities. Wesche *et al.* (2011) found that *U. pumila* seedlings in Mongolia are characterized by very low shoot–root ratios, which might indicate that seedlings need to reach water in the soil profile as soon as possible due to the dry conditions characteristic of these areas.

Our greenhouse experiment further revealed that the overall biomass production as well as shoot–root ratios increased with increasing water availability and warmer temperatures highlighting that water acquisition is of high importance for this species. However, we found that water and the temperature treatments had a stronger effect on populations from the native range than they had on non-native populations. While shoot–root ratios of populations from both non-native ranges showed only a moderate response to the temperature treatment, ratios of native populations clearly increased under warmer growing conditions (Fig. 5A). A similar strong increase of the shoot–root ratios of native populations was observed under the wet water treatment while non-native populations showed a weaker response (Fig. 5B). Non-native populations thus displayed lower levels of phenotypic plasticity than those from the native range. Siberian elm grows under very dry conditions in parts of its native range where it entirely depends on groundwater (Wesche *et al.* 2011). Plants growing in more stressful conditions (e.g. dry conditions) often reduce their biomass production while contributing more biomass to roots in order to balance the water absorption and consumption (van der Maarel and Titlyanova 1989; Wu *et al.* 2007).

Admittedly maternal effects might have also affected our results. These effects could be alleviated by using at least second-generation individuals for such comparative studies (Lavergne and Molofsky 2007; Moloney *et al.* 2009), but this approach is impractical for trees due to the long generation times. However, we assume that the overall enhanced growth performance and tolerance to different growth conditions of non-native *U. pumila* populations is rather connected to the apparently high genetic diversity and incidence of admixture in both non-native ranges (Cogolludo-Agustín *et al.* 2000; Zalapa *et al.* 2010; Hirsch *et al.* unpubl. data). Elevated genetic diversity often supports higher environmental tolerance of populations (Hodgins *et al.* 2012; Forsman and Wennersten 2015). According to our own data (Hirsch *et al.* unpubl. data), the high genetic diversity of the non-native populations of *U. pumila* likely resulted from multiple introductions that facilitated admixture of previously isolated populations. This genetic mixing and the resulting high genetic diversity levels could have facilitated the shift in early life cycle traits of *U. pumila* in the non-native ranges. Our assumption confirms the prediction that evolutionary processes in non-native species may occur rapidly because novel allelic combinations resulting from admixture can be beneficial in the face of new selective pressures (Barrett and Schluter 2008; Forsman 2014). It remains to be tested in future studies if these genetic shifts in non-native *U. pumila* populations are reflective of an adaptive evolutionary change. Therefore, reciprocal transplantation experiments under heterogeneous environmental conditions in both native and non-native ranges should be conducted to definitively infer the role of local adaptation in explaining performance differences (Huang *et al.* 2015; Gibson *et al.* 2016). Moreover, our results may be biased due to the sampling design representing relatively few geographical regions per range (Keller and Taylor 2008). Future experiments should, therefore, try to include more native as well non-native populations to confirm our findings over a wider geographical range.

Our findings also highlight the importance of considering early life cycle traits when implementing management against invasive populations. Specifically, for Siberian elm, our findings illustrate the need for management efforts to include early life stages to control further spread. Once *U. pumila* is established and a large amount of belowground biomass has accumulated, managing efforts become more difficult because the root-system must be destroyed to prevent resprouting (USDA 2014). The potential to establish rapidly new populations shown by our results implies that management efforts should, therefore, be focused on its early life stages to antagonize its further spread. 

## Conclusions

We used a combination of different methods covering various scales in our study to show that a shift in post-germination traits has likely occurred in non-native *U. pumila* populations. We assume that the enhanced early life cycle performance of non-native *U. pumila* populations is beneficial during establishment and colonization events across different growth conditions. This emphasizes the importance of considering potential post-introductory genetic shifts for predictions of invasion processes as well as for risk assessments for non-native species.

## Sources of Funding

This work was supported by the “Graduiertenfoerderung des Landes Sachsen-Anhalt” and by the German Academic Exchange Service (DAAD; Grant no. D/10/01844). Funding for the workshop was provided by the C•I•B, Stellenbosch University (through the office of the Vice Rector: Research, Innovation and Postgraduate Studies), and the South African National Research Foundation (DVGR Grant no. 98182).

## Contributions by the Authors

H.H. and H.v.W. formulated the idea. H.H., I.H., K.W., D.R. and H.v.W. designed the experiment. H.H. conducted fieldwork. H.H., C.W. and M.H. performed the greenhouse experiment. H.H., H.v.W. and M.H. analyzed the data. H.H. wrote the first draft of the manuscript and editorial advice was provided by all other authors.

## Conflict of Interest Statement

None declared.

## Supplementary Material

Supplementary Data
